# Tunable Transmission and Deterministic Interface states in Double-zero-index Acoustic Metamaterials

**DOI:** 10.1038/s41598-018-24773-6

**Published:** 2018-04-20

**Authors:** Wei Zhao, Yuting Yang, Zhi Tao, Zhi Hong Hang

**Affiliations:** 1 School of Electronic and Information Engineering, Soochow University, Suzhou, 215006 China; 2 College of Physics, Optoelectronics and Energy & Collaborative Innovation Center of Suzhou Nano Science and Technology, Soochow University, Suzhou, 215006 China; 3 Key Laboratory of Modern Optical Technologies (Soochow University), Education of Ministry & Key Lab of Advanced Optical Manufacturing Technologies of Jiangsu Province, Suzhou, 215006 China

## Abstract

Following the seminal work by Dubois *et al*. (*Nat. Commun*. **8**, 14871 (2017)), we study a double-zero-index acoustic metamaterial with triangular lattice. By varying the height and diameter of air scatterers inside a parallel-plate acoustic waveguide, acoustic dispersion of the first-order waveguide mode can be manipulated and various interesting properties are explored. With accidental degeneracy of monopolar and dipolar modes, we numerically prove the double-zero-index properties of this novel acoustic metamaterial. Acoustic waveguides with tunable and asymmetric transmission are realized with this double-zero-index acoustic metamaterial embedded. Band inversion occurs if the bulk acoustic band diagram of this acoustic metamaterial is tuned. Deterministic interface states are found to exist on the interface between two acoustic metamaterials with inverted band diagrams.

## Introduction

Metamaterials, artificial structures with exotic effective material properties, provide unprecedented manners to control waves, including light and sound. The flow of acoustic wave can be affected by material properties such as mass density *ρ* and bulk modulus *κ*. Resonating structures were experimentally developed to behave as a material with negative mass density^1^ or negative bulk modulus^2^, which are unavailable in nature. Ever since, research efforts have been devoted to acoustic metamaterial (AMM) designs to realize intriguing manipulations of acoustic waves, including negative refraction^3,4^, superlens and hyperlens to break diffraction limit^5–7^, cloaking^8–10^, parity-time acoustics^11,12^, space cancellation in complementary medium^13^, coherent perfect absorption of acoustic wave^14,15^, acoustic wavefront engineering using metasurface^16,17^, super absorption^18,19^ *et al*.

In the exploration of exotic *ρ* and *κ* using artificial structures, one type of AMMs, zero-index material (ZIM) was of particular interest^20–30^. The refractive index of AMM is defined as $\sqrt{\rho /\kappa }$. In principle, zero-index can be achieved with either effective mass density *ρ* approaching zero or effective bulk modulus *κ* approaching infinite at some frequencies, both of which are possible with resonating structures. Space-coiling metamaterials^21^ and zeroth-order Fabry-Perot mode^27^ were also utilized to behave as a near-zero refractive index. With no phase accumulation during propagation in a ZIM, acoustic wave can easily tunnel through an arbitrary waveguide^25^. However, double-zero-index (DZI) material, with simultaneous zero mass density and infinite bulk modulus is not easy. The distinct spatial symmetries related to effective mass density and bulk modulus^31^ make it challenging to design structures with effective *ρ* and *κ* resonating at the same frequency. Following the proposal using dielectric photonic crystals^32^, Dubois *et al*. suggested to realize an AMM with simultaneous zero mass density and infinite bulk modulus using a square array of cylindrical air scatterers in a two-dimensional (2D) waveguide whose height is larger than that of the background air channel^30^. Dirac-like cone dispersion at the Brillouin zone center appears on its acoustic band diagram of the first-order waveguide mode. The impedance of this DZI AMM was much improved compared to single-zero ones and a collimator of acoustic wave was achieved. Here, we extend the design principle of DZI AMMs to triangular lattice and various applications, including tunable and asymmetric transmission in an acoustic waveguide are demonstrated by finite element simulations. Moreover, we explore the tuning mechanism of the acoustic band diagrams of DZI AMMs. Acoustic band inversion is achieved with deterministic interface states realized numerically. With the successful demonstrations of previously reported applications in DZI materials, the effectiveness of DZI properties of the recently developed AMMs by Dubois *et al*. is further verified and we hope more efforts will be devoted to motivate applications in the new acoustic platform.

## Results

### Geometrical and theoretical statement of DZI acoustic metamaterial

**Figure 1:**
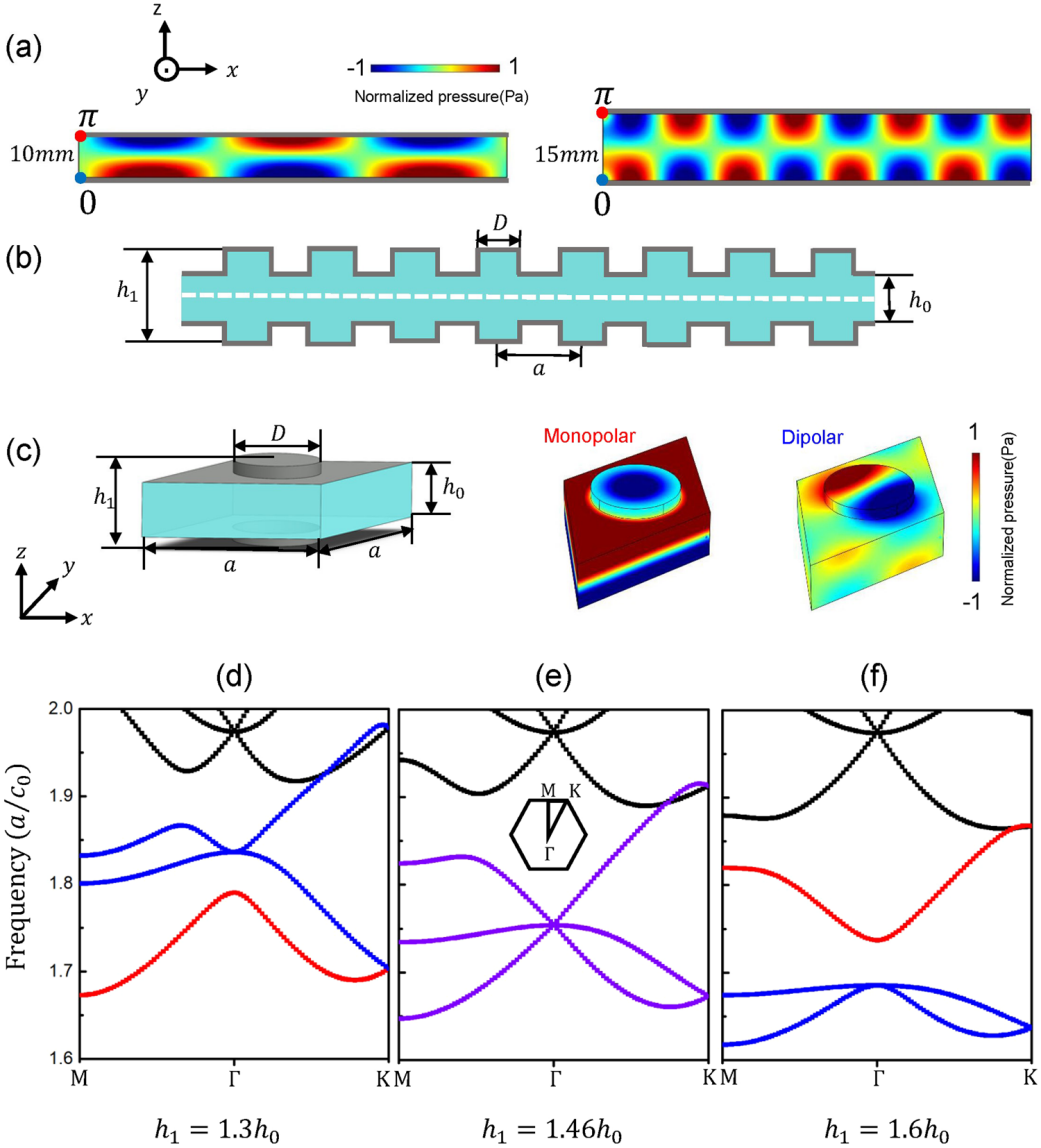
(**a**) The pressure field distributions of two acoustic parallel-plate waveguides with different heights: *h* = 10 *mm* (left) and *h* = 15 *mm* (right) at 18000 *Hz* for first-order waveguide mode. The red and blue dots represent line sources with identical amplitude but π phase difference. The gray lines represent hard wall boundaries. (**b**) Schematic of a waveguide with periodic variations of height. The dashed line indicates the soft boundary with zero pressure field, used to excite first-order waveguide mode. (**c**) The unit cell of an AMM in triangular lattice with *a* = 32 *mm*, *D* = 0.49375*a*, *h*_0_ = 0.3125*a* and *h*_1_ = 1.3*h*_0_ and its corresponding monopolar and dipolar eigen-mode profiles at *Γ* point. (**d**–**f**) Calculated band diagrams of AMMs with different heights *h*_1_ of air scatterers. The dispersion of monopolar mode and dipolar modes are indicated in red and blue respectively. The frequency of monopolar and dipolar modes can be configured by changing *h*_1_ and accidental triple-degeneracy with Dirac-like cone dispersion occurs (in purple) at *h*_1_ = 1.46*h*_0_.

Acoustic waves in air are longitudinal scalar waves. Longitudinal plane waves even at very low frequencies are allowed to propagate in a confined system. In a parallel-plate waveguide system, with two hard walls (in gray) parallel to each other as shown in Fig. 1a, there also exists waveguide modes at higher frequencies, whose pressure field amplitudes are maximized at both the hard wall boundaries and nodes exist inside the waveguide. For the first-order waveguide mode, the phases at the two hard wall boundaries differ by *π* and one node appears at the center of the waveguide. Thus, the wave number along the *z*-axis is ${k}_{z}=\pi /h$, where *h* is the height of the waveguide. We consider acoustic wave propagating along the *x*-axis and the corresponding wave vector is ${k}_{x}=\sqrt{\frac{{\omega }^{2}}{{c}_{0}^{2}}-\frac{{\pi }^{2}}{{h}^{2}}}$, where *ω* is the angular frequency of the acoustic wave and *c*_0_ = 343 *m*/*s* is the sound speed in air. It can be easily recognized that *h* shall be larger than half the working wavelength to allow acoustic wave of this waveguide mode to propagate. The phase velocity of the first-order waveguide mode is

1 $$c=\frac{\omega }{{k}_{x}}={(\sqrt{\frac{1}{{c}_{0}^{2}}-\frac{{\pi }^{2}}{{\omega }^{2}{h}^{2}}})}^{-1}$$

The height of waveguide *h* can be used to effectively control the phase velocity. We are showing the simulated pressure field distributions at 18000 *Hz* for different heights of waveguide *h* = 10 *mm* (left) and *h* = 15 *mm* (right) in Fig. 1a. In simulations, two line sources along *y* direction (indicated in dots) are positioned at the leftmost end of the waveguide with initial phase difference of π in order to solely excite the first-order waveguide mode. The pressure field is zero at the center of the waveguide. We truncate the right part of an infinitely long waveguide to have a better visualization of the mode profile. A waveguide with larger height *h* has a shorter effective acoustic wavelength, and thus a higher effective refractive index.

Waveguides with different heights can also be assembled periodically, as shown in Fig. 1b. The periodic variations of height of air channels are an analogy of a one-dimensional (1D) acoustic crystal with high-index (larger waveguide height *h*_1_) scatterers inside a low-index background (smaller background waveguide height *h*_0_). The refractive index contrast can be easily configured by the ratio between *h*_0_*/h*_1_. Moreover, strong scattering occurs due to the periodic arrangement of scatterers and the propagation properties of acoustic waves in such a 1D structure will be effectively altered by the filling ratio, namely *D/a*, where *D* is the width of air scatterer with height *h*_1_ and *a* is the lattice constant. Two independent geometrical parameters *h*_1_*/h*_0_ and *D/a* can be used to tune the acoustic dispersion of the first-order waveguide modes and the propagation of acoustic wave inside such a waveguide is thus controlled.

We extend to its 2D counterpart, with cylindrical scatterers of diameter *D* and height *h*_1_ periodically arranged in a triangular lattice within a waveguide of background height *h*_0_, whose unit cell can be found in the left panel of Fig. 1c. With six-fold rotational symmetry, the triangular lattice is more homogenous compared to previous study in square lattices and interesting properties are to be explored as follows. The acoustic band diagram is numerically obtained using finite element method as shown in Fig. 1d, with the parameters *a* = 32 *mm*, *D* = 0.496375*a*, *h*_0_ = 0.3125*a* and *h*_1_ = 1.3*h*_0_. The frequency is normalized to *a*/*c*_0_. In order to only excite the first-order waveguide modes, a virtual soft boundary with zero pressure field at the center of the waveguide is added during eigenmode analysis. A non-degenerate mode (in red, at lower frequency) and a double-degenerate mode (in blue, at higher frequency) exist at *Γ* point, the Brillion zone center. The non-degenerate and double-degenerate modes correspond to monopolar (Fig. 1c, mid panel) and dipolar (Fig. 1c, right panel) modes respectively. As the two degenerate dipolar modes are symmetric, only the profile of one mode is plotted. The monopolar mode modulates the effective bulk modulus and the dipolar mode affects the effective mass density of AMMs^33^. By increasing the height (*h*_1_) of scatterer, the frequencies of monopolar and dipolar modes approach each other and triple-degeneracy occurs at *h*_1_ = 1.46*h*_0_, as shown in Fig. 1e. Further increasing *h*_1_ will lead to a situation that monopolar mode is at a higher frequency than that of dipolar modes (Fig. 1f). The triple degeneracy as presented in Fig. 1e is an accidental degeneracy and Dirac-like cone dispersion will be guaranteed^34^. With only monopolar and dipolar interaction, the effective parameters close to Dirac-like cone can be retrieved to bear simultaneous zero mass density and infinite bulk modulus in acoustics^20,28,30^ and simultaneous zero permittivity and permeability in electromagnetism^32,35^. Because we have two independent parameters *h*_1_*/h*_0_ and *D/a* to tune the acoustic band diagram, the accidental degeneracy can be achieved in various parameters and DZI properties can be configured to different frequencies.

**Figure 2:**
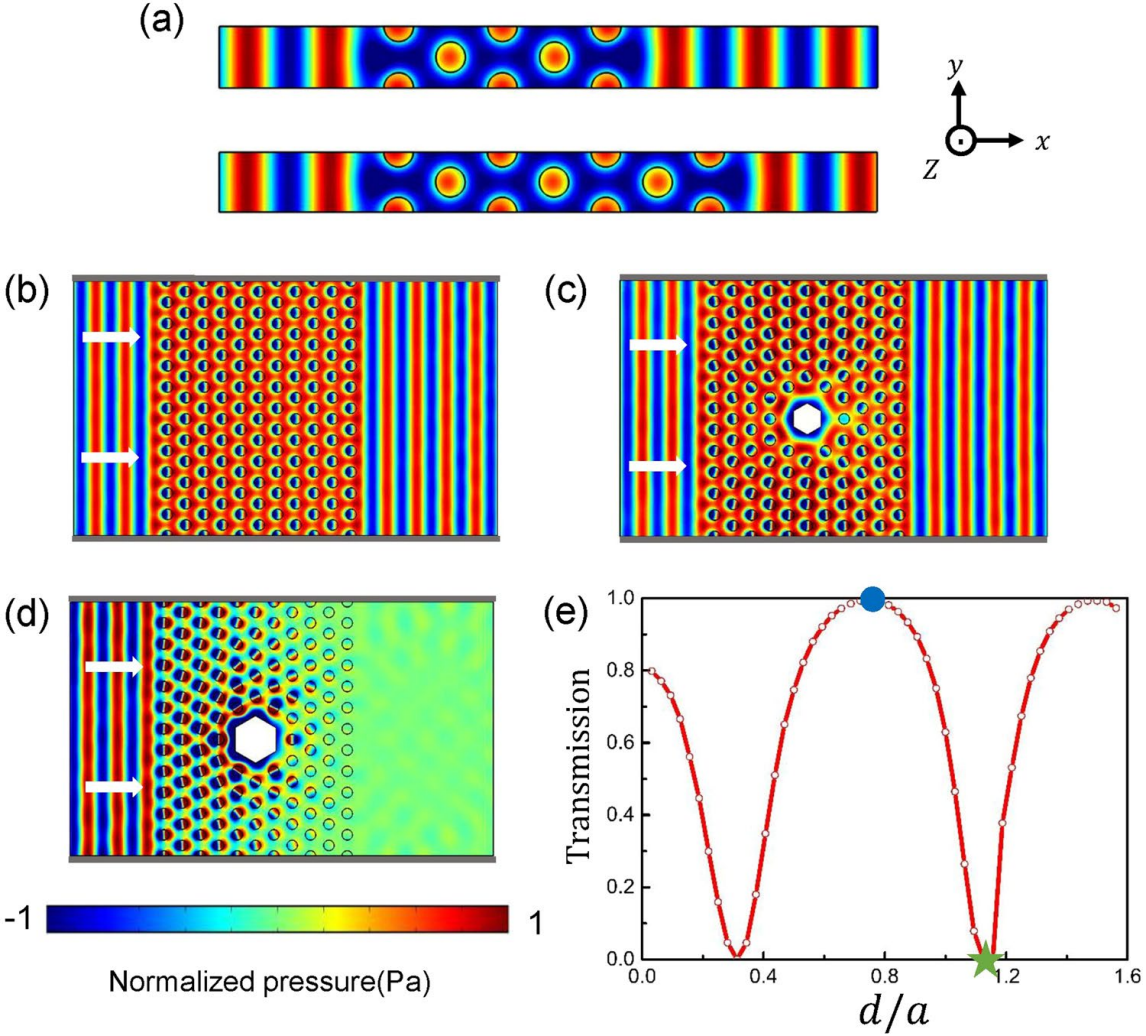
(**a**) Simulated pressure field distributions with plane wave incident to five and seven layers of designed DZI AMM at *f*_0_ = 1.755 *a*/*c*_0_ with periodic boundary conditions along *y* direction. (**b**) Total transmission through DZI AMM obtained by replacing periodic boundary condition with hard walls (indicated in gray) and increasing waveguide width. (**c**–**e**) The transmission through a waveguide can be effectively tuned by changing the size of an embedded hard defect. *d* is the side length of the hexagonal-shaped defect. The simulated pressure distributions corresponding to total transmission (blue dot, with *d* = *3/4a*) and total reflection (green star, with *d* = *9/8a*) are shown in (**c**) and (**d**) respectively.

We verify ZIM properties by numerical simulations. In Fig. 2a, waveguides with different numbers of air scatterers are simulated, at the working frequency *f*_0_ = 1.755 *a*/*c*_0_, which is the Dirac-like frequency in Fig. 1e. The width of the waveguide in *y* direction is *a*. Periodic boundary conditions are set on the *y*-direction and absorbing boundaries are applied at incident (left) and exit (right) end of the waveguide. Two line sources along *y* direction with same amplitude but π phase difference are placed on the two hard walls along *z* direction at the leftmost end of waveguide. The mass density and velocity of air are *ρ*_0_ = 1.21 *kg*/*m*^3^ and *c*_0_ = 343 *m/s*. The phases of acoustic waves after propagating through a waveguide with air scatterers are identical even though we change the thickness of acoustic metamaterials embedded to be $5\sqrt{3}/2a$ and $7\sqrt{3}/2a$, as shown in the upper and below panels of Fig. 2a respectively. Moreover, the pressure field inside the designed AMM is homogeneous in the *xy* plane. No phase accumulation occurs during propagation in such an AMM, which directly reflects its ZIM property. We further increase the width of waveguide to be much larger than the working wavelength, and the original periodic boundary condition along *y* direction in Fig. 2a is replaced by hard walls and the simulation result with acoustic wave emitting from left is shown in Fig. 2b. Though the width of waveguide is now 12*a*, the phase of acoustic wave is still homogenous inside, not to mention that the incident field is nearly identical to transmitted field. Total transmission can only be achieved through a single zero-index medium whose size is comparable to working wavelength in background but this limitation is removed for DZI medium, as discussed for electromagnetic wave^36^. Total transmission through a zero-index medium with such a size as presented here is an indirect proof that we have a DZI medium with the effective *ρ* and 1/*κ* approaching zero simultaneously where finite impedance can be obtained.

### The application of DZI acoustic metamaterial

We further verify the DZI properties by introducing some applications highly related to DZI properties to this novel AMM. Cloaking in a waveguide is also a property related to ZIM in both acoustics^23,24^ and electromagnetism^37^. Here, by removing the center twelve scatterers, a “cloaking region” with flat waveguide is created and some objects can be hidden inside. As shown in Fig. 2c, a hexagonal-shaped hard defect, with side length *d* = *3/4a*, is inserted at the center of cloaking region, connecting to both upper and bottom hard walls along *z* direction. Even with such a large scatterer embedded, nearly perfect transmission can be achieved with acoustic wave incidence at *f*_0_ = 1.755*a*/*c*_0_ and the phase of transmitted wave is identical to that without defect, indicating its ZIM property. We can further tune the transmission through a waveguide filled with DZI AMMs by changing the size of the hexagonal defect. Total transmission and reflection occurs with a defect of different side length embedded (Fig. 2e). Totally reflection is understandable. The scattering on the composite defect, including both the cloaking region and the hard defect may lead to zero pressure field at the boundary between DZI AMM and the cloaking region. As required by the homogeneity of pressure field inside a DZI AMM, the pressure field everywhere inside the DZI medium is forced to be zero and thus total reflection occurs, as shown in Fig. 2d with defect side length *d* = *9/8a*. We also note that similar tunable transmission properties were realized for elastic waves in simulations^29^. We need to clarify that the mechanism reported here is different. In ref.^29^, a fixed boundary is present between the embedded scatterer and ZIM. In other words, ZIM structure has to be changed accordingly when the size of the scatter is changed. To us, the tunablity achieved is not feasible in terms of applications. Here, as we only need to change the embedded scatterer in the fixed “cloaking” region but the ZIM structures are intact, the tunable transmission scheme we proposed is more favorable to experiments.

**Figure 3:**
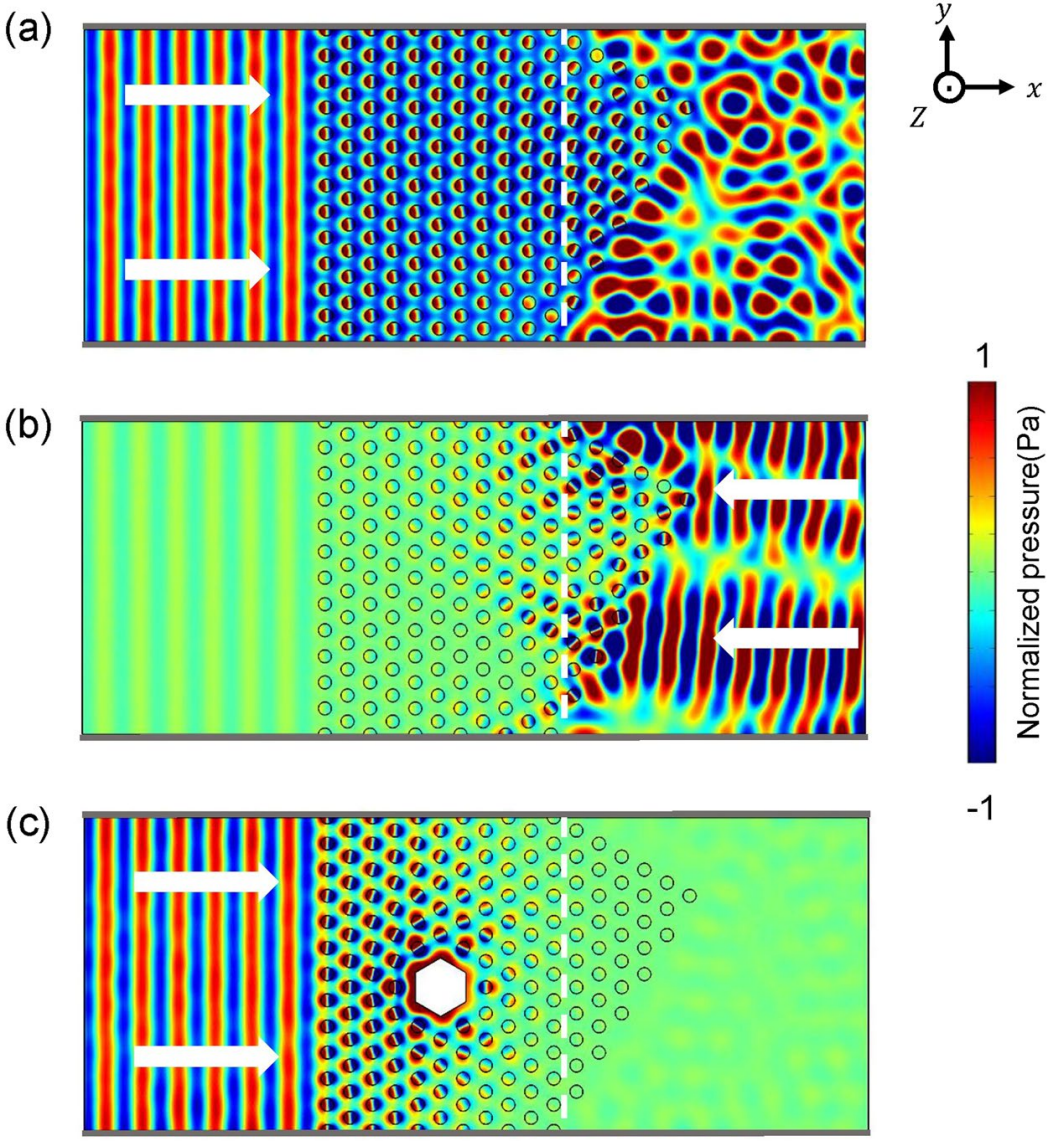
Pressure field distributions for a non-symmetric prism consisted of DZI AMMs for incident wave from left (**a**) and right (**b**) side at *f*_0_ = 1.755 *a*/*c*_0_. The gray lines represent hard wall boundaries. (**c**) Transmission from left can be suppressed if a hexagonal hard defect with side length *d* = *9/8a* is embedded.

An intriguing property of DZI material is that only normally incident wave can be coupled into it. Oblique incident wave will be totally reflected due to Snell’s law. By design, to include non-flat surfaces, asymmetric transmission can also be realized with the help of AMMs^26^. In Fig. 3, we consider another acoustic waveguide by attaching an acoustic prism fabricated using DZI AMMs to the waveguide discussed in Fig. 2. Now the AMM structure inside the waveguide is non-symmetric on the positive and negative *x* direction. All other parameters are identical to those as in Fig. 2. In Fig. 3a, plane wave at *f*_0_ = 1.755*a*/*c*_0_ incident from left impinges normally on the DZI AMM and can penetrate and pass through with high transmission. Contrarily, if the plane wave is incident from right, it will be almost totally reflected back as shown in Fig. 3b. We note that as triangular lattice is considered, the prism can be of different surface truncations (for instance 30 and 60 degree interfaces) while only 45 degree truncation as for a square lattice is possible in ref.^26^. As the coupling to DZI material is still possible at the vertex of the prism, small portion of acoustic energy is transmitted leftward. More interestingly, a tunable asymmetric acoustic device can also be realized using the structure we propose. The transmission from left can be suppressed if a hexagonal hard defect of side length *d* = *9/8a* is inserted, and the simulated pressure field distribution is shown in Fig. 3c. As discussed in Fig. 2d, the waveguide now is cut-off for acoustic wave incidence from both sides. Tunable transmission is also possible upon a change of the side length of the hard defect.

### Deterministic interface states

Topology has cast new inspiration to acoustics. Topological acoustics^38–45^ arouse research interest to use classical wave systems to verify theories predicted in condensed matter physics. Geometrical phase enters classical acoustics as Xiao and co-workers observed a confinement of acoustic wave at the interface separating two 1D acoustic crystals with inverted band properties^38^. As the existence of interface states is solely determined by the bulk acoustic band properties, they are denoted as deterministic interface states. The design to these deterministic interface states is simple and thus topology/geometrical phase can motivate novel design principle to acoustic equipment. It is preferable to study interface states at 2D or higher dimensions, because acoustic wave propagation can thus be controlled by these interface states. Whether DZI AMM can be used to design interface states leave a good question to be explored.

**Figure 4:**
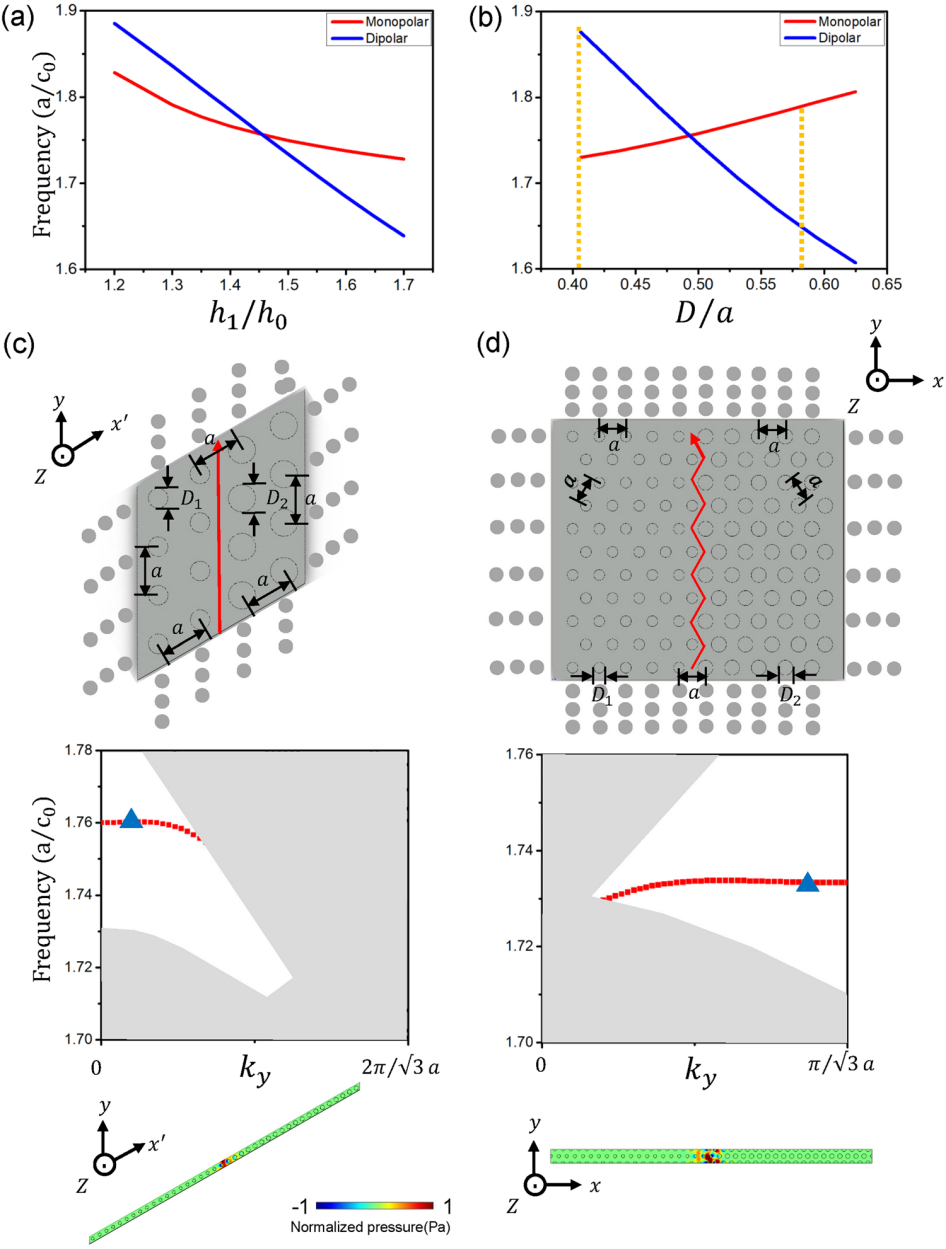
(**a**) and (**b**) Phase diagram of monopolar and dipolar modes with the height (**a**) and diameter (**b**) of air scatterer changing. (**c**) and (**d**) The existence of deterministic interface states when band inversion of monopolar and dipolar modes occurs. Two configurations, with *Γ* − *K* (**c**) and *Γ* − *M* (**d**) orientation are considered. Top panels: The schematics of two interface connection between AMM1 with *D*_1_ = 0.40625*a* and AMM2 with *D*_2_ = 0.5625*a*. *h*_0_ = 0.3125*a* and *h*_1_ = 1.46*h*_0_ are used for both AMM1 and AMM2. Mid panels: Calculated acoustic band diagram with a super cell with twenty unit cells of AMM1 (AMM2) left (right) to the interface (along y axis). The folding of first-order waveguide modes belonging to AMM1 or AMM2 are painted in gray and the deterministic interface state is denoted in red squares. Bottom panels: Calculated eigen pressure field distributions at frequency 1.76 *a*/*c*_0_ with ${k}_{y}=0.2\frac{2\pi }{\sqrt{3}a}$ for *Γ* − *K* orientation and at frequency 1.7336 *a*/*c*_0_ with ${k}_{y}=0.8\frac{\pi }{\sqrt{3}a}$ for *Γ* − *M* orientation, as indicated by solid triangles in mid panels.

DZI properties are induced by the intriguing Dirac-like cone dispersion at Brillion zone center, triggered by accidental degeneracy of monopolar and dipolar modes of acoustic dispersion. We have witnessed that by changing the height of air channels, the frequencies of the monopolar and dipolar modes can be inverted. We plot the frequencies of monopolar modes and dipolar modes at *Γ* point at different heights of scatterers within a background waveguide with height *h*_0_ = 0.3125*a*, as shown in Fig. 4a. Band inversion occurs at *h*_1_ = 1.46*h*_0_, where triple-degeneracy and Dirac-like cone appears. The monopolar and dipolar modes are tied to different spatial symmetries: from Fig. 1b, monopolar mode is symmetric while dipolar mode is anti-symmetric. Thus the swop of mode frequency with different symmetries infers that there exists a phase transition of bulk acoustic band properties^46^. As both the height variation (*h*_1_*/h*_0_) and filling ratio (*D/a*) can be used to effectively tune the acoustic dispersion of the first-order waveguide mode, it is no wonder that similar phase diagram appears as presented in Fig. 4b that band inversion occurs when increasing the diameter *D* of the scatterers.

To numerically verify the different bulk acoustic band properties, we select two structures with *D*_1_ = 0.40625*a* (referred as AMM1) and *D*_2_ = 0.5625*a* (referred as AMM2), while fixing *h*_0_ = 0.3125*a* and *h*_1_ = 1.46*h*_0_ and connect these two AMMs together. AMM1 and AMM2 belong to before and after the phase transition respectively (indicated as dashed lines in Fig. 4b). Their bulk band property shall be different if an interface state appears immediately at their interface. As the band inversion occurs at the Brillion zone center, we can consider two totally different interfacial connections, along *Γ* − *K* and *Γ* − *M* directions as for the triangular lattice we used, whose illustration can be found in the top panels of Fig. 4c and d respectively. We numerically obtained the acoustic band diagrams of a supercell with twenty unit cells of AMM1 (AMM2) left (right) to the interface (along *y* axis) as shown in the mid panel of Fig. 4c, whose corresponding unit cell structure can be found in the bottom panel. Periodic boundary conditions are applied on both *y* and *x′* directions. Rhombic unit cell is considered and the two base vectors of the unit cell are not orthogonal to each other. A soft boundary with zero pressure is artificially set at the center plane of the waveguide structure and thus only odd order of waveguide mode can be excited. In order to guide eyes, the folding of the first-order waveguide modes belonging to AMM1 or AMM2 are painted in gray and an extra band, existing in the common gap between AMM1 and AMM2 appears in the calculated band diagram, as denoted in red squares. Eigen mode analysis is also carried out where the eigen pressure field distribution at the frequency 1.76 *a*/*c*_0_, corresponding to ${k}_{y}=0.2\frac{2\pi }{\sqrt{3}a}$ is shown in the bottom panel of Fig. 4c, as indicated by a triangle in the mid panel. Strong confinement to the interface between AMM1 and AMM2 are observed. Similarly, we carried out similar investigation on the *Γ* − *M* orientation and very similar phenomena are achieved (as shown in Fig. 4d). Twenty layers of AMM1 and AMM2 are adopted in the supercell calculation and periodic boundary conditions are now applied on both *y* and *x* directions. Please be noted that as the super cell under investigation is not its primitive cell, the calculated interface dispersion (denoted in red in the mid panel of Fig. 4d) does not start from Brillion zone center.

**Figure 5:**
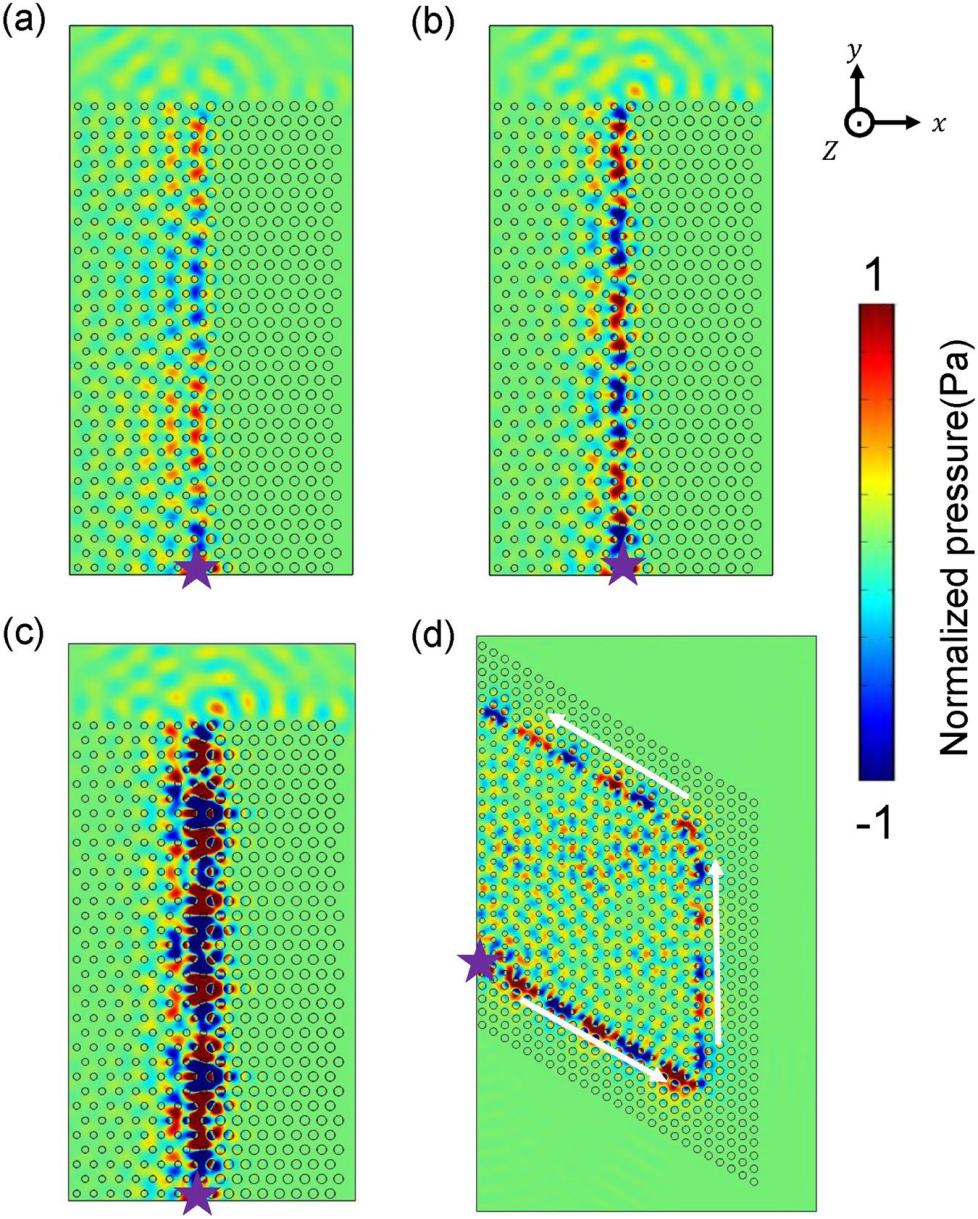
The simulated pressure field distributions excited by two point sources (violet stars) with identical amplitude and π phase difference to excite first-order waveguide mode. (**a**–**c**) The simulated deterministic interface states with different vectors at frequencies 1.730, 1.733, 1.734 *a*/*c*_0_ with *Γ* − *M* orientation connection between AMM1 and AMM2. (**d**) The deterministic interface state at frequency 1.758 *a*/*c*_0_ can turn 60° and 120° along the interface with *Γ* − *K* orientation connection.

The existence and propagation properties of the deterministic interface states are also investigated by numerical simulations. In Fig. 5, we simulate the excitation of deterministic interface states in different geometries. As we are interested in the first-order waveguide modes, a pair of point sources (whose positions indicated by violet stars) is placed on the upper and bottom hard wall boundaries along the *z* directions of the waveguide, with identical amplitudes but π phase difference. In Fig. 5a–c, we consider *Γ* − *M* orientation, with AMM1 and AMM2 are assembled on the left and right to the source. At different frequencies, 1.730 *a*/*c*_0_ (Fig. 5a), 1.733 *a*/*c*_0_ (Fig. 5b) and 1.734 *a*/*c*_0_ (Fig. 5c), the interface states are well excited and good confinement to the interface is observed. Moreover, the pattern of interface state pressure field distributions repeats itself and its repeat rate is different at different frequencies, indicating their different propagating wave vectors. We also construct a rhombus AMM1 structure and surround it with five layers of AMM2 structure, as shown in Fig. 5d. This configuration corresponds to the *Γ* − *K* orientation connection between AMM1 and AMM2, which is unavailable in the study of square lattice^46^. Some interesting properties can be observed. The propagation of interface state at 1.758 *a*/*c*_0_ is not disturbed ever though sharp turns of 60° and 120° are included in the interface we construct.

## Conclusions

In conclusion, we follow the study of double-zero-index acoustic metamaterial^30^ and extend to verify its double-zero-index properties using triangular lattice. With six-fold rotational symmetry, triangular lattice is intrinsically more homogenous than four-fold rotationally symmetric square lattice and we hope this new lattice will help with the future design using double-zero-index acoustic metamaterials. We explore some applications tightly related to the double-zero-index properties of this novel type of acoustic metamaterial. The transmission through an acoustic waveguide filled by double-zero-index medium can be effectively tuned if the size of the embedded hard defect in the fixed cloaking region is changed. By further including a prism with surface not perpendicular to incident acoustic wave, asymmetric but tunable transmission can be achieved with one side of the waveguide turnoff while the sound flow can be controlled with acoustic wave incident from the other. We study in details the tuning mechanism of the acoustic dispersion of this type of acoustic metamaterial. The frequencies of the symmetric monopolar mode and antisymmetric dipolar modes can be inverted, inferring a phase transition. Two phase diagrams related to the bulk acoustic band properties are provided and we verify the inverted band property by the realization of a deterministic interface state existing on the interface between two acoustic metamaterials inside different regions of the phase diagram. Two orientations of interfacial connections are investigated and our numerical simulations suggest that deterministic interface states existing in double-zero-index acoustic metamaterials could benefit future acoustic equipment design.

## Methods

Throughout this paper, all numerical simulations are performed by **COMSOL** Multiphysics, 5.3, commercial software based on finite element method (www.comsol.com).

### Data availability

The data in this study are available from the authors upon reasonable request.
